# Optimal Criteria and Diagnostic Ability of Serum Pepsinogen Values for *Helicobacter pylori* Infection

**DOI:** 10.2188/jea.JE20170094

**Published:** 2019-04-05

**Authors:** Shogo Kikuchi, Mototsugu Kato, Katsuhiro Mabe, Takashi Kawai, Takahisa Furuta, Kazuhiko Inoue, Masanori Ito, Masaharu Yoshihara, Masaaki Kodama, Kazunari Murakami

**Affiliations:** 1 Department of Public Health, Aichi Medical University, School of Medicine, Aichi, Japan; 2 Division of Endoscopy, Hokkaido University Hospital, Sapporo, Japan; 3 Department of Cancer Preventive Medicine, Graduate School of Medicine, Hokkaido University, Sapporo, Japan; 4 Department of Gastroenterological Endoscopy, Tokyo Medical University, Tokyo, Japan; 5 Center for Clinical Research, Hamamatsu University School of Medicine, Shizuoka, Japan; 6 Department of General Medicine, Kawasaki Medical School, Okayama, Japan; 7 Department of Gastroenterology and Metabolism, Hiroshima University, Hiroshima, Japan; 8 Health Service Center, Hiroshima University, Hiroshima, Japan; 9 Department of Gastroenterology, Faculty of Medicine, Oita University, Oita, Japan; 10 National Hospital Organization Hakodate Hospital, Hakodate, Hokkaido, Japan; 11 Department of Gastroenterology, National Hospital Organization Hakodate Hospital, Hakodate, Hokkaido, Japan; 12 Center for Gastroenterological Endoscopy, Asahigaoka Hospital, Okayama, Japan

**Keywords:** *Helicobacter pylori*, infection status, serum pepsinogen, diagnostic accuracy, gastric cancer

## Abstract

**Background:**

Practical criteria for the use of serum pepsinogen (PG) values in diagnosing *Helicobacter pylori* infection have not yet been determined.

**Methods:**

The results of gastric endoscopies, *H. pylori* infection tests, and PG values were retrospectively reviewed. Subjects were assigned to groups, including never-infected (with neither infection nor gastric mucosal atrophy), infected (with atrophy or findings indicating infection in endoscopy and positive infection tests except for antibody tests), and ex-infected (with gastric mucosal atrophy and negative infection tests, except for antibody tests). The optimal criteria with combined use of the PG II concentrations and the PG I/PG II ratio were investigated separately for PG measurements obtained with the chemiluminescent magnetic particle immunoassay (CLIA) and latex agglutination (LA) methods, such that the specificity was greater than 70% and the sensitivity was no less than 95% among the never-infected and infected subjects. Similar analyses were performed by combining the data from ex-infected and infected subjects.

**Results:**

For the CLIA (LA) method, the optimal criterion among 349 (397) never-infected and 748 (863) infected subjects was a PG II value of at least 10 (12) ng/mL or a PG I/PG II ratio no more than 5.0 (4.0), which produced 96.3% (95.1%) sensitivity and 82.8% (72.8%) specificity. When 172 (236) ex-infected subjects were included, the optimal criterion was the same, and the sensitivity was 89.1% (86.9%).

**Conclusions:**

The above criteria may be practical for clinical use, and PG tests using these criteria might prevent unnecessary endoscopic examinations for never-infected subjects.

## INTRODUCTION

The risk of gastric cancer is substantially different depending on an individual’s *Helicobacter pylori* infection status. Subjects without a history of infection (never-infected) have very low risk, while subjects with persistent infection (infected) have a high risk. Infected subjects have a risk of gastric cancer that is at least 20 times as high as never-infected subjects.^1^^,^^2^

PG reflects gastric mucosal atrophy and inflammation.^3^^–^^5^ In the 1980s in Japan, the prevalence of *Helicobacter pylori* was over 80% among individuals over 40 years of age,^6^ and both the incidence and mortality of gastric cancer was very high. Thus, a search was conducted for a marker that reflects the risk of gastric cancer.^7^^,^^8^ Because gastric cancer risk is positively correlated with the severity of gastric mucosal atrophy, PG came to be used as a marker of gastric cancer risk among individuals harboring *H. pylori*.^7^ A practical criterion that diagnosed the severity of gastric mucosal atrophy and consequently indicated a high risk of gastric cancer was established and has been used since it was developed.^9^

Nevertheless, the prevalence of *H. pylori* infection has been decreasing^6^^,^^10^ in Japan. Among those who are 50–59 years old, the prevalence was approximately 70% in 1990,^11^ and it was 50% in 2010.^10^ As mentioned above, the risk of gastric cancer is very different between individuals with and without *H. pylori* infection. Therefore, it becomes more important to diagnose whether a subject is harboring *H. pylori* or not rather than to diagnose the severity of gastric mucosal atrophy. If the risk of gastric cancer can be evaluated through serum tests, subjects without a history of *H. pylori* infection, who have low risk, can avoid burden of unnecessary examinations. Thus, a new way to use PG measurements has been proposed, which is to distinguish between individuals with and without *H. pylori* infection.^12^^,^^13^

There are subjects with a past history of infection (ex-infected) who have experienced successful eradication therapy or auto-disappearance of *H. pylori*. Successful eradication reduces gastric cancer risk,^14^^,^^15^ while subjects who experience auto-disappearance have a risk of gastric cancer similar to infected subjects.^16^ Even after successful eradication, gastric cancer risk is so high that it is an indicator in endoscopic examinations.^17^^,^^18^ In ex-infected subjects, those without a memory or history of eradication therapy were included. Therefore, the goal was for the PG test to distinguish never-infected subjects from infected or ex-infected subjects as a marker of *H. pylori* infection considering gastric cancer risk.

Although several studies have shown the usefulness of the PG test as a marker of *H. pylori* infection,^13^^,^^19^ practical criteria for determining infection status have not been established. To determine the practical criteria and evaluate the diagnostic ability, data were collected retrospectively from subjects with test results from gastric endoscopic examinations, *H. pylori* infection tests, and PG values.

## SUBJECTS AND METHODS

### Study population

The subjects were adult patients who received gastrointestinal endoscopic examinations, *H. pylori* infection tests (at least one of the following: urea breath test, stool antigen test, rapid urease test, histological examination, and culture of a biopsied specimen), and PG tests using the chemiluminescent magnetic particle immunoassay (CLIA) or latex agglutination (LA) methods at Hokkaido University Hospital, Tokyo Medical University Hospital, Kawasaki Medical University Hospital, Central Hospital, Heisei-Kurashiki Hospital, Hiroshima University Hospital or Oita University Hospital from January 2006 through October 2014. Subjects with current proton pump inhibitor use, severe renal failure, autoimmune gastritis, a history of successful *H. pylori* eradication therapy, and/or gastrectomy were excluded. Individuals with insufficient data were also excluded. All subjects were included no matter their diagnosis unless the exclusion criteria were met.

### Diagnosis of *H. pylori* infection status

Histological atrophy of gastric mucosa is well correlated with endoscopic findings,^20^^,^^21^ atrophy of gastric mucosa was observed far more frequently in subjects with *H. pylori* infection than individuals without,^22^ and endoscopic atrophy rarely disappear after successful *H. pylori* eradication.^23^ Thus, ex-infected subjects were distinguished from never-infected subjects by observing gastric mucosal atrophy through endoscopy. A recent study showed that endoscopic examination effectively distinguishes never-infected subjects from other subjects.^24^

A subject was classified as never-infected if he/she had no apparent history of *H. pylori* infection, showed little atrophy (C-0 or C-1 on the Kimura-Takemoto endoscopic classification^20^), and had negative results in all performed *H. pylori* infection tests, including serum antibody tests. A subject was classified as infected if he/she showed atrophy or findings indicating infection in endoscopic examination^25^ and positive results in at least one of the following tests: urea breath test, stool antigen test, rapid urease test, histological examination, or culture of a biopsied specimen. A subject was classified as ex-infected if he/she showed atrophy (C-2 or more on the endoscopic classification) and negative results on all performed *H. pylori* infection tests, except for antibody tests. Antibody test, which gives positive results during some duration after disappearance of *H. pylori*, was used only in diagnosis of a never-infected subject.

### Statistical analyses and selection of optimal criteria

We assume never-infected subjects with diagnoses of normal stomach or gastritis as “healthy subjects” and calculated the mean and 2.5, 25, 50, 75 and 97.5 percentiles of PG I, PG II, and PG I/PG II values using their data. Never-infected subjects with gastritis were included, because the clinical diagnosis “gastritis” is often used for subjects with little gastric lesion who undergo endoscopic examination, and the border with normal stomach is not clear. As the “healthy subjects” have low risk of both gastric cancer and peptic ulcer diseases, they need not undergo endoscopic examination if they have no symptoms.

The diagnostic ability of the serum PG test to distinguish between never-infected and infected subjects was evaluated. The analyses were conducted separately for results obtained using two methods to measure serum PG values, including the CLIA (“ARCHITECT pepsinogen I, II, Abbott”; Abbott Co. Ltd., Tokyo, Japan) and LA (“L-Z test, Eiken”; Eiken Chemical Co. Ltd., Tokyo, Japan) methods. Then, ex-infected subjects without a history of *H. pylori* eradication therapy were added to the group of infected subjects, and similar analyses were performed. These additional analyses were performed because ex-infected patients without a memory or history of eradication therapy, who have high risk of gastric cancer, are included among the subjects of the PG tests.

The diagnostic abilities of the PG I, PG II, and PG I/PG II values were compared for never-infected and infected subjects using receiver operating characteristic (ROC) curves. Candidate criteria using two of the three values (PG I, PG II or PG I/PG II) were also applied, and the sensitivity and specificity, as well as the positive and negative likelihood ratios and their 95 percent confidence intervals (CIs), were calculated. We considered sensitivity to be superior and decided preferable diagnostic accuracy occurred at 95% sensitivity and 70% specificity among subjects because false-negative results provoke severe effects in the risk evaluation of gastric cancer, especially in exclusion of subjects with low risk. We selected the optimal criteria of tests showing the preferable diagnostic accuracy, considering the balance between sensitivity and specificity with superiority on sensitivity. To evaluate the influence of ex-infected subjects, ex-infected subjects without a history of *H. pylori* eradication were added to the group of infected subjects and similar analyses were performed. Furthermore, analyses were also performed in which never-infected subjects were restricted to those with normal stomach or gastritis, who need not to undergo endoscopic examination.

This study was approved by the Ethics Board of Aichi Medical University, School of Medicine.

## RESULTS

For the CLIA method, data from 1,674 subjects were collected. Of those subjects, 405 were excluded, and 1,269 were eligible for the study (52.7% were male, and the mean and median ages were 56.0 [standard deviation {SD}, 15.5] and 58 years, respectively). For the LA method, data from 1,981 subjects were collected. Among those subjects, 485 were excluded, and 1,496 were eligible for the study (64.5% were male, and the mean and median ages were 59.6 [SD, 14.9] and 62 years, respectively). The subjects with PG values obtained by the CLIA method included 349 never-infected, 748 infected, and 172 ex-infected subjects. The subjects with PG values obtained by the LA method included 397 never-infected, 863 infected, and 236 ex-infected subjects. These details are shown in Table 1, and the clinical diagnoses of eligible subjects are shown in Table 2.

**Table 1.** Numbers of and details for collected, included, and excluded subjects

Method	CLIA			LA		
Collected (Total)	1,674	(100%)		1,981	(100%)	

Included (Subtotal)	1,269	(75.8%)	(100%)	1,496	(75.5%)	(100%)
Never-infected	349 (320^a^)		(27.5%)	397 (347^a^)		(26.5%)
Infected	748		(58.9%)	863		(57.7%)
Ex-infected	172		(13.6%)	236		(15.8%)

Excluded (Subtotal)	405	(24.2%)	(100%)	485	(24.5%)	(100%)
PPI use	85		(21.0%)	261		(53.8%)
Successful eradication	45		(11.1%)	74		(15.3%)
PPI or Eradication	254		(62.7%)	0		(0.0%)
Type A gastritis	1		(0.2%)	1		(0.2%)
History of gastrectomy	2		(0.5%)	2		(0.4%)
Insufficient data	18		(4.4%)	147		(30.3%)

CLIA: chemiluminescent magnetic particle immunoassay; LA, latex agglutination.

^a^Of never infected subjects, those with diagnosis of normal stomach or gastritis were assumed as healthy subjects as shown in Table 2.

**Table 2.** Clinical diagnoses of the subjects used in the analyses

Infection status	Normal stomach	Gastritis	Peptic ulcer	Adenoma	Gastric cancer	Lymphoma of stomach	Other GI disease^a^	Extra-gastric diseases	Unknown	Total
CLIA method										
Never-infected	266 (76.2%)^b^	54 (15.5%)^b^	12 (3.4%)	0 (0.0%)	7 (2.0%)	1 (0.3%)	7 (2.0%)	1 (0.3%)	1 (0.3%)	349 (100%)
Infected	3 (0.4%)	346 (46.3%)	192 (25.7%)	22 (2.9%)	155 (20.7%)	21 (2.8%)	5 (0.7%)	3 (0.4%)	1 (0.1%)	748 (100%)
Ex-infected	8 (4.7%)	64 (37.2%)	34 (19.8%)	5 (2.9%)	53 (30.8%)	4 (2.3%)	4 (2.3%)	0 (0.0%)	0 (0.0%)	172 (100%)
Total	277 (21.8%)	464 (36.6%)	238 (18.8%)	27 (2.1%)	215 (16.9%)	26 (2.0%)	16 (1.3%)	4 (0.3%)	2 (0.2%)	1,269 (100%)
LA method										
Never-infected	97 (24.4%)^b^	250 (63.0%)^b^	9 (2.3%)	1 (0.3%)	13 (3.3%)	1 (0.3%)	25 (6.3%)	0 (0.0%)	1 (0.3%)	397 (100%)
Current	2 (0.2%)	482 (55.9%)	176 (20.4%)	20 (2.3%)	149 (17.3%)	18 (2.1%)	11 (1.3%)	4 (0.5%)	1 (0.1%)	863 (100%)
Ex-infected	2 (0.8%)	120 (50.8%)	5 (2.1%)	20 (8.5%)	85 (36.0%)	1 (0.4%)	3 (1.3%)	0 (0.0%)	0 (0.0%)	236 (100%)
Total	101 (6.8%)	852 (57.0%)	190 (12.7%)	41 (2.7%)	247 (16.5%)	20 (1.3%)	39 (2.6%)	4 (0.3%)	2 (0.1%)	1,496 (100%)

CLIA, chemiluminescent magnetic particle immunoassay; GI, gastrointestinal; LA, latex agglutination.

^a^Other gastrointestinal diseases.

^b^Of never infected subjects, those with diagnosis of normal stomach or gastritis were assumed as healthy subjects. In some analyses and Table 3, healthy subjects were used instead of never-infected subjects.

In Table 3, the percentiles of PG values in the never-infected subjects are shown as values in the “healthy subjects”. Distributions of PG I and PG II values in the subjects were nearly normal after logarithmic transformation was applied. The geometric mean of PG I and PG II values obtained using the CLIA method were 47.8 (95% CI, 46.0–49.7) and 6.70 (95% CI, 6.40–7.00) ng/mL, respectively. Values for the LA method were 54.3 (95% CI, 52.2–56.5) and 9.75 (95% CI, 9.37–10.15) ng/mL, respectively. Distributions of PG I to PG II ratio were nearly normal. Arithmetic means using the CLIA and the LA methods were 7.32 (SD, 1.65) and 5.78 (SD, 1.59), respectively.

**Table 3.** Percentiles and geographic/arithmetic means for serum pepsinogen values in the healthy subjects^a^

	Percentiles					Geographic/arithmetic mean (95% confidence interval)
	2.5	25	50 (Median)	75	97.5	
CLIA method (*n* = 320)						
PG I (ng/mL)	24.6	37.7	47.3	59.1	84.0	47.8 (46.0–49.7)^b^
PG II (ng/mL)	2.90	5.30	6.70	8.30	14.63	6.70 (6.40–7.00)^b^
PG I/PG II	4.62	6.11	7.25	8.38	11.18	7.32 (7.14–7.50)^c^

LA method (*n* = 347)						
PG I (ng/mL)	27.6	42.8	51.5	67.2	150.7	54.7 (52.5–57.1)^b^
PG II (ng/mL)	5.00	7.60	9.50	11.80	26.94	9.77 (9.37–10.18)^b^
PG I/PG II	3.66	4.80	5.50	6.50	9.33	5.78 (5.61–5.95)^c^

CLIA, chemiluminescent magnetic particle immunoassay; LA, latex agglutination; PG I, pepsinogen I; PG II, pepsinogen II; PG I/II, pepsinogen I to II ratio.

^a^Healthy subjects: never-infected subjects with diagnosis of normal stomach or gastritis as shown in Table 2.

^b^Geometric mean.

^c^Arithmetic mean.

**Figure 1.**
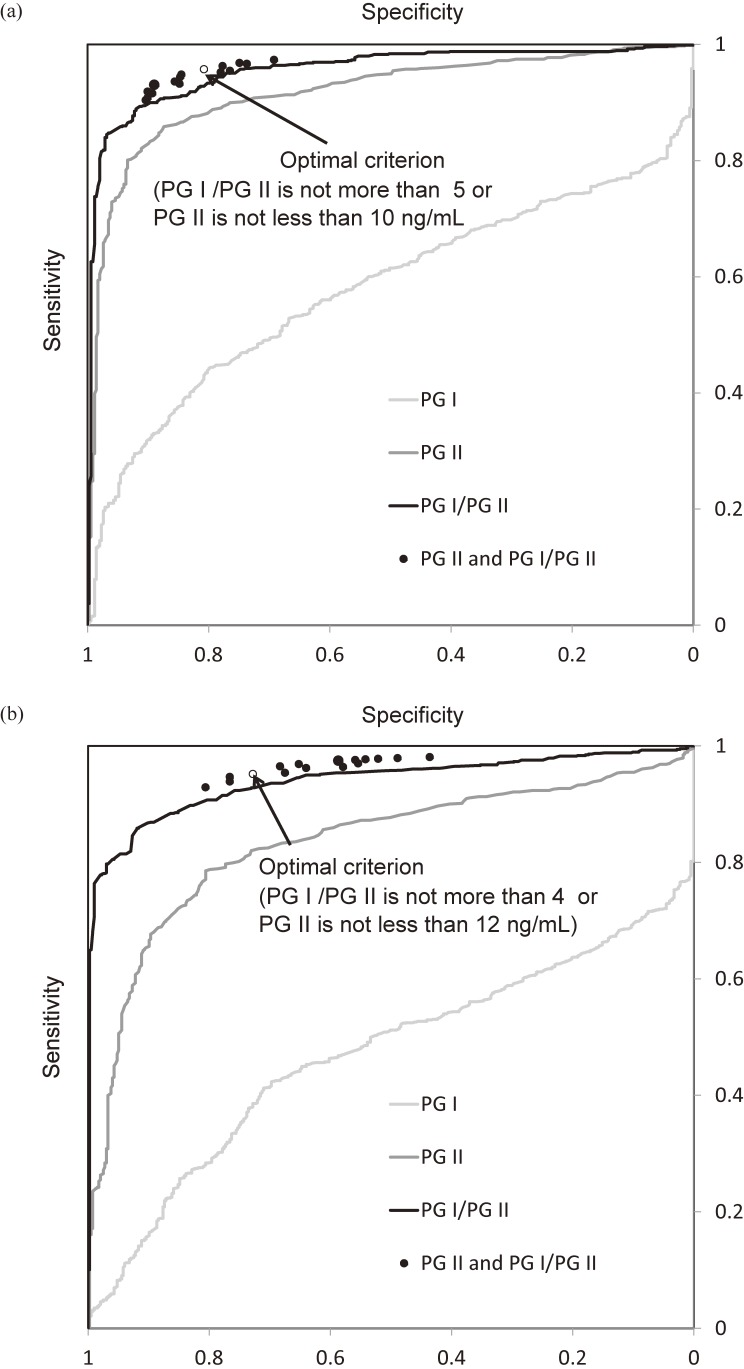
Specificities and sensitivities for *H. pylori* infection under the criteria for serum pepsinogen (PG) values are shown. Ex-infected subjects are not included. The label 1a indicates the CLIA method and 1b indicates the LA method. The results of PG I (less than a value defined as positive), PG II (greater than a positive value) and PG I to PG II ratio (PG I/PG II, less than a positive value) are expressed as receiver operating characteristic curves, and the combinations of PG II and PG I/PG II values are indicated with circles. The optimal one is marked by an open circle and the others are closed circles. It is clear that the criteria of the combinations produce better accuracy than separate PG I, PG II or PG I/PG II values do.

In the ROC curve analyses of the never-infected and infected subjects determined using the CLIA method, the areas under the curves for PG I, PG II, and PG I/PG II were 0.579, 0.917, and 0.955, respectively, and the optimistic cut-off values for PG II and PG I/PG II were 11.4 ng/mL (sensitivity: 80.1% and specificity: 93.4%) and 4.61 (sensitivity: 84.5% and specificity: 96.8%), respectively. In the corresponding analyses of subjects with values obtained using the LA method, the areas under the curves for PG I, PG II, and PG I/PG II were 0.470, 0.832, and 0.939, respectively, and the optimal cut-off values for PG II and PG I/PG II were 12.5 ng/mL (sensitivity: 78.9% and specificity: 79.6%) and 4.11 (sensitivity: 85.9% and specificity: 91.9%), respectively (Figure 1).

According to the results of the analyses, PG II and PG I/PG II were indicated as useful markers for diagnosis of *H. pylori* infection. Optimal criteria for *H. pylori* infection diagnosis using a combination of PG II and PG I/PG II values were investigated using never-infected and infected subjects, with values obtained from the CLIA and LA methods analyzed separately. Infection was defined as positive when the PG II value was not less than the cutoff value or the PG I/PG II ratio was not higher than the cutoff value. In the analyses of the CLIA method, the cutoff values were set at 9, 10, 11, or 12 ng/mL for PG II and at 4, 4.5, 5, or 5.5 for PG I/PG II. For the LA method, the cutoff values were set at 10, 11, 12, or 13 for PG II and at 3.5, 4, 4.5, or 5 for PG I/PG II. These results are shown in Table 4 and Table 5.

**Table 4.** Specificity and sensitivity for *H. pylori* infection under each criterion for measures obtained with the CLIA method

Criterion^a^		Specificity (95% CI)^b^	Ex-infected patients not included^c^			Ex-infected patients included^d^		
		
PG II	PG I/II		Sensitivity (95% CI)^b^	LR+ (95% CI)	LR− (95% CI)	Sensitivity (95% CI)^b^	LR+ (95% CI)	LR− (95% CI)
9	4.5	78.8 (74.5–83.1)	96.0 (94.6–97.4)	4.53 (4.51–4.55)	0.051 (0.048–0.054)	87.9 (85.8–90.0)	4.15 (4.13–4.17)	0.153 (0.151–0.156)
9	5	75.6 (71.1–80.1)	97.1 (95.8–98.3)	3.99 (3.97–4.00)	0.039 (0.036–0.042)	90.2 (88.3–92.1)	3.70 (3.69–3.72)	0.129 (0.127–0.132)
9	5.5	70.8 (66.0–75.5)	97.7 (96.7–98.8)	3.34 (3.33–3.36)	0.032 (0.029–0.036)	91.3 (89.5–93.1)	3.12 (3.11–3.14)	0.123 (0.120–0.126)
10	3.5	87.7 (84.2–91.1)	93.2 (91.4–95.0)	7.56 (7.52–7.60)	0.078 (0.075–0.081)	83.5 (81.1–85.9)	6.78 (6.73–6.82)	0.188 (0.186–0.191)
10	4	87.1 (83.6–90.6)	93.9 (92.1–95.6)	7.28 (7.24–7.32)	0.071 (0.068–0.074)	84.8 (82.5–87.1)	6.58 (6.54–6.61)	0.175 (0.172–0.177)
10	4.5	86.8 (83.3–90.4)	95.1 (93.5–96.6)	7.21 (7.17–7.25)	0.057 (0.054–0.060)	86.6 (84.4–88.8)	6.57 (6.54–6.61)	0.154 (0.152–0.156)
10	5	82.8 (78.8–86.8)	96.3 (94.9–97.6)	5.60 (5.57–5.63)	0.045 (0.042–0.048)	89.1 (87.1–91.1)	5.18 (5.16–5.21)	0.131 (0.129–0.134)
10	5.5	76.5 (72.1–81.0)	97.1 (95.8–98.3)	4.13 (4.11–4.15)	0.038 (0.035–0.042)	90.3 (88.4–92.2)	3.84 (3.83–3.86)	0.126 (0.124–0.129)
11	4	91.1 (88.1–94.1)	92.1 (90.2–94.0)	10.37 (10.31–10.43)	0.087 (0.084–0.089)	82.5 (80.0–85.0)	9.29 (9.23–9.35)	0.192 (0.190–0.194)
11	4.5	90.8 (87.8–93.9)	93.6 (91.8–95.3)	10.21 (10.15–10.26)	0.071 (0.068–0.073)	84.6 (82.2–86.9)	9.22 (9.17–9.28)	0.170 (0.168–0.172)
11	5	86.5 (83.0–90.1)	95.2 (93.7–96.7)	7.07 (7.03–7.10)	0.056 (0.053–0.059)	87.5 (85.4–89.6)	6.50 (6.46–6.53)	0.144 (0.142–0.147)
11	5.5	79.4 (75.1–83.6)	96.4 (95.1–97.7)	4.67 (4.65–4.69)	0.045 (0.042–0.049)	89.2 (87.2–91.2)	4.33 (4.30–4.35)	0.136 (0.133–0.138)
12	4	92.6 (89.8–95.3)	90.8 (88.7–92.8)	12.18 (12.11–12.25)	0.100 (0.097–0.102)	81.0 (78.4–83.5)	10.87 (10.80–10.94)	0.206 (0.204–0.207)
12	4.5	92.3 (89.5–95.1)	92.5 (90.6–94.4)	11.96 (11.89–12.03)	0.081 (0.079–0.084)	83.4 (81.0–85.8)	10.78 (10.71–10.84)	0.180 (0.178–0.182)
12	5	88.0 (84.6–91.4)	94.1 (92.4–95.8)	7.82 (7.78–7.86)	0.067 (0.064–0.070)	86.4 (84.2–88.6)	7.18 (7.14–7.22)	0.154 (0.152–0.157)
12	5.5	79.7 (75.4–83.9)	95.7 (94.3–97.2)	4.71 (4.68–4.73)	0.054 (0.051–0.057)	88.5 (86.4–90.5)	4.35 (4.33–4.37)	0.145 (0.142–0.147)

CI, confidence interval; CLIA, chemiluminescent magnetic particle immunoassay, PG II, pepsinogen II; PG I/II, pepsinogen I to II ratio; LR+, positive likelihood ratio; LR−, negative likelihood ratio.

^a^Defined as positive when PG II (ng/mL) was no less than this value or PG I/II was no more than this value.

^b^Percent.

^c^Sensitivity was calculated using 748 infected patients. LR+ and LR− were calculated using both the 748 patients and 349 never-infected patients.

^d^Sensitivity was calculated using 748 infected and 172 ex-infected patients. LR+ and LR− were calculated using both the 748+172 patients and 349 never-infected patients.

The optimal criterion was a PG II value of 10 ng/mL or greater or a PG I/PG II ratio of 5.0 or less (shaded). See text for details of its selection.

**Table 5.** Specificity and sensitivity for *H. pylori* infection under each criterion for measures obtained with the LA method

Criterion^a^		Specificity (95% CI)^b^	Ex-infected patients not included^c^			Ex-infected patients included^d^		
		
PG II	PG I/II		Sensitivity (95% CI)^b^	LR+ (95% CI)	LR− (95% CI)	Sensitivity (95% CI)^b^	LR+ (95% CI)	LR− (95% CI)
10	3.5	57.9 (53.1–62.8)	96.3 (95.0–97.6)	2.29 (2.28–2.30)	0.064 (0.060–0.068)	88.5 (86.7–90.4)	2.10 (2.10–2.11)	0.198 (0.194–0.201)
10	4	55.4 (50.5–60.3)	96.9 (95.7–98.0)	2.17 (2.17–2.18)	0.056 (0.052–0.061)	89.8 (88.0–91.6)	2.01 (2.01–2.02)	0.184 (0.180–0.188)
10	4.5	52.1 (47.2–57.1)	97.7 (96.7–98.7)	2.04 (2.04–2.05)	0.044 (0.040–0.049)	91.8 (90.2–93.4)	1.92 (1.91–1.92)	0.157 (0.153–0.161)
10	5	43.6 (38.7–48.5)	98.0 (97.1–99.0)	1.74 (1.73–1.74)	0.045 (0.040–0.051)	93.7 (92.3–95.2)	1.66 (1.66–1.66)	0.144 (0.139–0.149)
11	3.5	67.5 (62.9–72.1)	95.2 (93.8–96.7)	2.93 (2.92–2.94)	0.070 (0.067–0.074)	86.7 (84.7–88.7)	2.67 (2.66–2.68)	0.197 (0.194–0.200)
11	4	64.0 (59.3–68.7)	96.1 (94.8–97.4)	2.67 (2.66–2.68)	0.062 (0.058–0.065)	88.4 (86.5–90.2)	2.45 (2.44–2.46)	0.182 (0.179–0.185)
11	4.5	58.7 (53.8–63.5)	97.3 (96.3–98.4)	2.36 (2.35–2.36)	0.045 (0.042–0.050)	90.8 (89.1–92.5)	2.20 (2.19–2.21)	0.157 (0.153–0.160)
11	5	48.9 (43.9–53.8)	97.8 (96.8–98.8)	1.91 (1.91–1.92)	0.045 (0.041–0.050)	93.2 (91.7–94.7)	1.82 (1.82–1.83)	0.140 (0.136–0.144)
12	3.5	76.6 (72.4–80.7)	93.7 (92.1–95.4)	4.00 (3.99–4.02)	0.082 (0.079–0.085)	84.7 (82.6–86.8)	3.62 (3.60–3.63)	0.200 (0.197–0.202)
12	4	72.8 (68.4–77.2)	95.1 (93.7–96.6)	3.50 (3.48–3.51)	0.067 (0.064–0.070)	86.9 (84.9–88.9)	3.19 (3.18–3.21)	0.180 (0.178–0.182)
12	4.5	65.2 (60.6–69.9)	96.8 (95.6–97.9)	2.78 (2.77–2.79)	0.050 (0.046–0.053)	89.9 (88.1–91.7)	2.59 (2.58–2.60)	0.155 (0.152–0.158)
12	5	54.2 (49.3–59.1)	97.6 (96.5–98.6)	2.13 (2.12–2.13)	0.045 (0.041–0.049)	92.6 (91.1–94.2)	2.02 (2.01–2.03)	0.136 (0.133–0.140)
13	3.5	80.6 (76.7–84.5)	92.8 (91.1–94.5)	4.79 (4.76–4.81)	0.089 (0.086–0.092)	83.3 (81.1–85.6)	4.30 (4.28–4.32)	0.207 (0.205–0.209)
13	4	76.6 (72.4–80.7)	94.6 (93.0–96.1)	4.04 (4.02–4.05)	0.071 (0.068–0.074)	85.9 (83.8–88.0)	3.67 (3.65–3.68)	0.184 (0.182–0.186)
13	4.5	68.3 (63.7–72.8)	96.4 (95.2–97.6)	3.04 (3.03–3.05)	0.053 (0.049–0.056)	89.3 (87.4–91.1)	2.81 (2.80–2.82)	0.157 (0.155–0.160)
13	5	55.9 (51.0–60.8)	97.5 (96.4–98.5)	2.21 (2.20–2.22)	0.046 (0.042–0.050)	92.4 (90.8–93.9)	2.10 (2.09–2.10)	0.137 (0.133–0.140)

CI, confidence interval; LA, latex agglutination; PG II, pepsinogen II; PG I/II, pepsinogen I to II ratio; LR+, positive likelihood ratio; LR−, negative likelihood ratio.

^a^Defined as positive when PG II (ng/mL) was no less than this value or PG I/II was no more than this value.

^b^Percent.

^c^Sensitivity was calculated using 863 infected patients. LR+ and LR− were calculated using both the 863 patients and 397 never-infected patients.

^d^Sensitivity was calculated using 863 infected and 236 ex-infected patients. LR+ and LR− were calculated using both the 863+236 patients and 397 never-infected patients.

The optimal criterion was decided so that sensitivity was more than 95% and it produced the best specificity among subjects while excluding the ex-infected ones. It was a PG II value of 12 ng/mL or greater or a PG I/PG II ratio of 4.0 or less (shaded).

For the CLIA method, nine criteria showed greater than 95% sensitivity and greater than 70% specificity. Among the three criteria showing more than 80% specificity, the two (having a PG II value of 10 ng/mL and a PG I/PG II ratio of 4.5 and with PG II: 11 and PG I/PG II: 5.0) with more than 86% specificity gave inferior sensitivity to the three criteria (PG II: 9 and PG I/PG II: 5.0 or 5.5 and PG II: 10 and PG I/PG II: 5.5) with more than 97% sensitivity. The other criterion (PG II: 10 and PG I/PG II: 5.0) with 82.8% specificity gave 96.3% sensitivity, which was not remarkably inferior to any other criteria except one (PG II: 9 and PG I/PG II: 5.5), with 97.7% sensitivity and 70.8% specificity. Considering these results, we selected the criterion with a PG II value of 10 ng/mL or a PG I/PG II ratio of 5.0 as the optimal one, which produced 96.3% (95% CI, 94.9–97.6%) sensitivity and 82.8% (95% CI, 78.8–86.8%) specificity, as well as positive and negative likelihood ratios of 5.60 (95% CI, 5.57–5.63) and 0.045 (95% CI, 0.042–0.048), respectively. This criterion produced the largest positive likelihood ratios of all criteria, along with negative likelihood ratios less than 0.05. For the LA method, a criterion with a PG II value of 12 ng/mL or higher or a PG I/PG II ratio of 4.0 or lower produced 95.1% (95% CI, 93.7–96.6%) sensitivity and 72.8% (95% CI, 68.4–77.2%) specificity, as well as positive and negative likelihood ratios of 3.50 (95% CI, 3.48–3.51) and 0.067 (95% CI, 0.064–0.070), respectively. This criterion produced the largest positive likelihood ratio of all criteria, along with negative likelihood ratios less than 0.07. The other criteria did not satisfy diagnostic accuracy requirements of 95% sensitivity and 70% specificity.

In the analyses when ex-infected subjects were included, 4–10% decreases in sensitivity were observed compared to the analyses that did not include this group (Table 4 and Table 5), and 36–76% of ex-infected subjects were diagnosed as positive with these criteria (data not shown). For the CLIA method, the sensitivity, specificity, positive likelihood ratio, and negative likelihood ratio were 89.1% (95% CI, 87.1–91.1%), 82.8% (95% CI, 78.8–86.8%), 5.18 (95% CI, 5.16–5.21), and 0.131 (95% CI, 0.129–0.134), respectively, under the optimal criterion determined when the ex-infected subjects were not included. This criterion produced the highest specificity among the criteria, resulting in greater than 88% sensitivity, which may be optimal when ex-infected subjects were included. The optimal criterion produced the largest positive likelihood ratio of all criteria, showing negative likelihood ratios no greater than 0.150.

For the LA method, the sensitivity, specificity, positive likelihood ratio, and negative likelihood ratios were 86.9% (95% CI, 84.9–88.9%), 72.8% (95% CI, 68.4–77.2%), 3.19 (95% CI, 3.18–3.21), and 0.180 (95% CI, 0.178–0.182), respectively, under the optimal criterion determined when the ex-infected subjects were excluded. This criterion produced the highest specificity among the criteria, with greater than 86% sensitivity, which may be optimal when ex-infected subjects are included. Among the criteria leading to negative likelihood ratios no greater than 0.180, the optimal one produced the largest positive likelihood ratios. Thus, there was no discrepancy between the results for sensitivity and specificity and the results for positive and negative likelihood ratios.

When never-infected subjects were restricted to those with normal stomach or gastritis, the same criteria as the analyses without the restriction were selected as the optimal ones both for CLIA and LA methods. For the CLIA method, the same nine criteria showed greater than 95% sensitivity and greater than 70% specificity, and the selection of the optimal criterion was similar. For the LA method, only the same criterion showed greater than 95% sensitivity and greater than 70% specificity. Specificity and positive and negative likelihood ratios were 85.0% (95% CI, 81.1–88.9%), 6.42 (95% CI, 6.38–6.45) and 0.044 (95% CI, 0.041–0.047), respectively for the CLIA method, while they were 72.0% (95% CI, 67.3–76.8%), 3.40 (95% CI, 3.39–3.42) and 0.068 (95% CI, 0.064–0.071), respectively for the LA method. When ex-infected subjects were included, positive and negative likelihood ratios were 5.94 (95% CI, 5.91–5.98) and 0.128 (95% CI, 0.126–0.130), respectively, for the CLIA method and 3.11 (95% CI, 3.09–3.12) and 0.182 (95% CI, 0.179–0.184), respectively, for the LA method. These results were similar to the results without the restriction of never-infected subjects.

## DISCUSSION

The optimal criteria that could distinguish never-infected subjects from infected subjects were investigated. The optimal criterion for values obtained with the CLIA method was a PG II concentration no less than 10 ng/mL or a PG I/PG II ratio no more than 5.0, while the criterion for data obtained with the LA method was a PG II concentration no less than 12 ng/mL or a PG I/PG II ratio no more than 4.0. The results determined using sensitivity and specificity were consistent with the results using likelihood ratios. The optimal criteria, as well as the PG values in the never-infected subjects, differed depending on the method of serum PG measurement. Differences in measurement results seemed to exist between these two methods,^19^ and the criteria for the practical use of serum PG values should be determined separately. In the current study, sera from 399 subjects were measured with both CLIA and LA methods. Differences in the mean of PG I, PG II values, and PG I/II ratio were 1.2, 2.7 (CLIA<LA), and 0.45 (CLIA>LA), respectively, where *P*-values by paired *t*-test were all less than 0.01. The difference in measured values between the two methods may be the main reason for the different optimal criteria, although difference of subjects could exert a little influence. One of the aims of using the serum PG test is to avoid unnecessary endoscopic or contrast X-ray examinations of the upper gastrointestinal tract for never-infected subjects. The effect may be reduced if specificity is low. However, low sensitivity results in missing *H. pylori* infected/ex-infected subjects with high risk of gastric cancer and may increase advanced gastric cancer with poor prognoses through delayed diagnosis. Because missing of subjects with high gastric cancer risk is thought to be more serious than an increase in unnecessary examinations, we decided that preferable diagnostic accuracy occurred at 95% sensitivity and 70% specificity among subjects. In the selection of the optimal criterion for the CLIA method, nine candidate criteria satisfied the preferable diagnostic accuracy. Considering the balance between sensitivity and specificity with superiority on sensitivity, we selected the optimal one among the three criteria giving more than 80% specificity.

A specificity of 70–80% indicates that 20–30% of never-infected subjects may undergo unnecessary endoscopic examinations after a serum PG test using the corresponding criteria. Although the expected burden of unnecessary examinations is not negligible, the serum PG test may allow 70% of never-infected subjects to avoid these tests. The prevalence of *H. pylori* infection is decreasing, and the number of never-infected subjects is increasing in Japan,^6^^,^^10^ which may increase the usefulness and importance of the serum PG test in the future.

The serum PG test showed approximately 95% sensitivity under the optimal criteria in the analyses that excluded ex-infected subjects, so it is thought to be a useful test for *H. pylori* infection. Nevertheless, the sensitivity decreased to approximately 88% when ex-infected subjects were included in the analyses because only 57–58% of ex-infected subjects met the criteria. The ex-infected subjects included in the analyses were those who had past *H. pylori* infection but did not have the infection when examined. Serum PG reflects both inflammation and atrophy of the gastric mucosa.^3^^,^^4^ Ex-infected subjects may have atrophy but not inflammation of the gastric mucosa at the time of serum and endoscopic examinations, which could be responsible for the lower positive rate. Ex-infected subjects include those with auto-disappearance of *H. pylori* due to the progression of severe gastric mucosal atrophy,^25^^–^^27^ those with unintended eradication due to antibiotics used to treat another disease, and those who underwent successful eradication therapy but do not remember receiving treatment. The frequency of unintended eradication reflects the frequency of antibiotic use for other diseases. The frequency of patients without memory of eradication therapy can be reduced through sufficient explanations at the time of eradication therapy and through careful interviews conducted immediately before the PG test. Thus, the frequency of ex-infected subjects may be influenced via artificial factors and may differ depending on clinics/hospitals and possibly doctors, as well as locations and populations in Japan. It seems inappropriate to automatically include ex-infected subjects in these analyses when PG values are used to determine the criteria to distinguish never-infected subjects from infected subjects for generalized use. Instead, analyses with and without these subjects should be performed. Fortunately, the optimal criteria did not differ between the analyses conducted with and without ex-infected subjects in the current study, and the results are thought to be considerably robust.

Practically, it is necessary for never-infected subjects with such diagnoses as gastric cancer or peptic ulcer diseases to receive endoscopic examination, while it is unnecessary for never-infected subjects with diagnoses of normal stomach or gastritis. We assumed the latter subjects to be “healthy subjects” and calculated percentiles and geometric/arithmetic means (Table 3). When the “healthy subjects” were used in the analyses instead of all never-infected subjects, the same optimal criteria were selected and specificity showed tiny changes from 82.8% to 85.0% for the CLIA method and from 72.8% to 72.0% for the LA method, which may indicate that the results are stable.

A study with 276 never-infected and 80 infected subjects showed that a PG II concentration of 9.9 ng/mL and a PG I/PG II ratio of 5.0 were separate optimal cutoff values for measurements obtained with the CLIA method for *H. pylori* infection.^10^ Another study investigated the optimal criteria for measurements obtained with the CLIA method from 19 never-infected and 291 infected subjects, as well as measurements obtained with the LA method with 158 never-infected and 2,365 infected subjects. Both studies indicated the optimal values were a PG II value of no less than 10 ng/mL or a PG I/PG II ratio of no more than 5.0.^23^ The optimal criteria identified in the current study were similar to these studies. Although a small difference in results was found regarding the LA method, the results of the current study with 397 never-infected and 863 infected subjects may be more stable. Thus, the criteria established in the current study may be reliable and have a practical use. As gastric cancer and peptic ulcer diseases are rare among never-infected subjects of Japanese general population,^28^^–^^30^ the criteria for the PG tests using the CLIA and LA methods may allow approximately 83% and 72% of the never-infected subjects, respectively, to avoid unnecessary endoscopic examinations, as the specificities indicate, while it may provoke approximately 4–5% (11–13% when ex-infected subjects are included) false-negative results in infected or ex-infected subjects of the population, who actually have to receive endoscopic examinations.

The current study is retrospective, and most subjects were outpatients of university hospitals who had been referred from other hospitals or clinics. As shown in Table 2, subjects with severe gastric diseases, including gastric cancer, were frequent compared with general population and outpatients. Thus, some sampling bias could exist. However, subjects with drugs or diseases that affected PG values were excluded from the analyses, as well as the inclusion or exclusion of each subject. Although this exclusion may minimize the sampling bias, combined with the relatively large sample size of the current study, attention should be paid to the external validity in practical use of the results.

In conclusion, for measurements obtained using the CLIA method, the criterion of a PG II concentration no less than 10 ng/mL or a PG I/PG II ratio no more than 5.0, as well as a criterion of a PG II concentration no less than 12 ng/mL or a PG I/PG II ratio no more than 4.0 for measurements obtained using the LA method, produced optimal diagnostic accuracy to identify *H. pylori*-infected subjects. The specific criteria for the PG tests may considerably reduce unnecessary endoscopic examinations, while they provoke some false-negative results. Sufficient explanations during eradication therapy and careful interviews conducted immediately before PG tests are necessary to minimize the frequency of false-negative results for ex-infected subjects.
